# Delphinidin Increases the Sensitivity of Ovarian Cancer Cell Lines to 3-bromopyruvate

**DOI:** 10.3390/ijms22020709

**Published:** 2021-01-12

**Authors:** Natalia Pieńkowska, Grzegorz Bartosz, Paulina Furdak, Izabela Sadowska-Bartosz

**Affiliations:** 1 Laboratory of Analytical Biochemistry, Institute of Food Technology and Nutrition, College of Natural Sciences, Rzeszow University, 4 Zelwerowicza Street, 35-601 Rzeszow, Poland; natalia.pien@gmail.com (N.P.); paulinaf2@o2.pl (P.F.); 2 Department of Bioenergetics, Food Analysis and Microbiology, Institute of Food Technology and Nutrition, College of Natural Sciences, Rzeszow University, 4 Zelwerowicza Street, 35-601 Rzeszow, Poland; gbartosz@ur.edu.pl

**Keywords:** ovarian cancer, anthocyanidins, delphinidin, glycolytic inhibitors, 3-bromopyruvate

## Abstract

3-Bromopyruvic acid (3-BP) is a promising anticancer compound. Two ovary cancer (OC) cell lines, PEO1 and SKOV3, showed relatively high sensitivity to 3-BP (half maximal inhibitory concentration (IC_50_) of 18.7 and 40.5 µM, respectively). However, the further sensitization of OC cells to 3-BP would be desirable. Delphinidin (D) has been reported to be cytotoxic for cancer cell lines. We found that D was the most toxic for PEO1 and SKOV3 cells from among several flavonoids tested. The combined action of 3-BP and D was mostly synergistic in PEO1 cells and mostly weakly antagonistic in SKOV3 cells. The viability of MRC-5 fibroblasts was not affected by both compounds at concentrations of up to 100 µM. The combined action of 3-BP and D decreased the level of ATP and of dihydroethidium (DHE)-detectable reactive oxygen species (ROS), cellular mobility and cell staining with phalloidin and Mitotracker Red in both cell lines but increased the 2’,7’-dichlorofluorescein (DCFDA)-detectable ROS level and decreased the mitochondrial membrane potential and mitochondrial mass only in PEO1 cells. The glutathione level was increased by 3-BP+D only in SKOV3 cells. These differences may contribute to the lower sensitivity of SKOV3 cells to 3-BP+D. Our results point to the possibility of sensitization of at least some OC cells to 3-BP by D.

## 1. Introduction

The term “ovarian cancer” (OC) includes several different types of cancer that all arise from cells of the ovary. Most commonly (in nearly 90%), tumors arise from the epithelium of the ovary [1]. The American Cancer Society estimates that, in 2020, about 21,750 American women will be diagnosed with OC, and 13,940 women will die from the disease. The initial treatment for advanced-stage OC frequently includes a surgical staging or debulking procedure, followed by a combination of platinum and Taxol adjuvant chemotherapy [2,3]. Despite a high response rate to first-line chemotherapy, the majority of women with advanced stage OC will relapse, with the median progression free survival being 16 months after initial diagnosis. Further, the five-year survival is dismal at less than 40% [4,5]. Therefore, new treatment strategies are urgently needed to improve outcomes in women with OC. 

**Figure 1:**
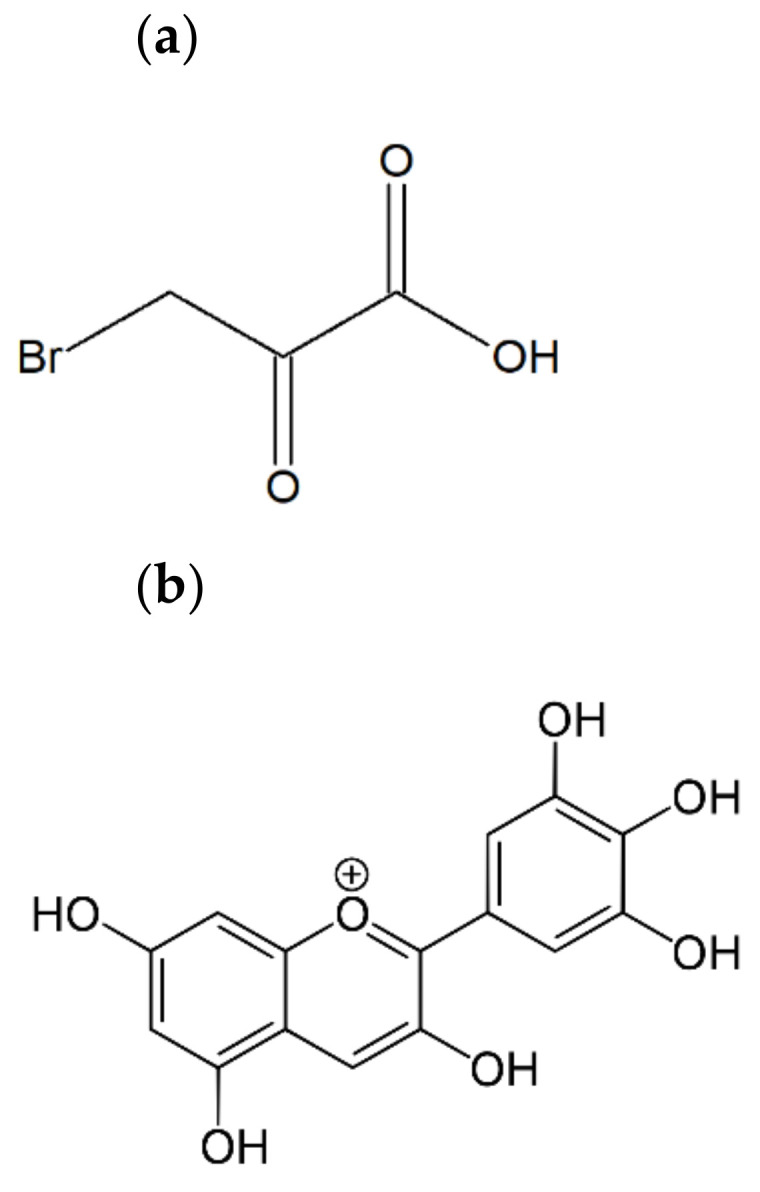
The chemical structure of 3-bromopyruvic acid (**a**) and delphinidin (3,3′,4′,5,5′,7-hexahydroxyflavylium) (**b**).

Most, if not all, cancer cells rely to a greater degree on glycolysis than normal cells. This phenomenon is referred to as the “Warburg effect” [6]. Since glycolysis is a much poorer energy source than oxidative phosphorylation, cancer cells have a significantly higher (about 10-fold higher than normal cells) demand for glucose. The Warburg effect enabling the survival of cancer cells in an oxygen-poor environment may be their Achilles heel, allowing for their eradication with a considerable degree of selectivity. Nevertheless, cancer cells can produce energy also from other sources. The extent of domination of energy metabolism by glycolysis is variable among cancers of various origin and cancerous cell lines, and oxidative metabolism remains functional in most glycolytic cancer cells [7,8]. Glycolytic inhibitors—among them, 3-bromopyruvic acid (3-BP; Figure 1a)—were proven to inhibit effectively the growth of cancer cell lines and malignant tumors [9,10,11]. Recently, we studied the effect of 3-BP on the mobility and survival of rat and human prostate cancer metastatic cell lines and revealed a high sensitivity of these lines to 3-BP, exceeding the sensitivity of other cell lines studied, with noticeable effects even at 5–10-μM 3-BP [12]. The literature [13] and our own data show that OC cells can show such a high sensitivity to 3-BP as well. However, further sensitization of cancer ovary cells to 3-BP would be desirable.

Anthocyanins, a subfamily of flavonoids, are consumed in fruits and vegetables, mostly in berries, grapes, pomegranates, red cabbage and sweet potatoes, as well as red wine [14,15], and belong to the most commonly applied natural food supplements because of their desirable coloring properties and potential health benefits [16]. Delphinidin (D; Figure 1b) belongs to the most popular anthocyanidins (aglycons of anthocyanins). 

Anthocyanidins, among other flavonoids, have been reported to be cytotoxic to cancer cells. Delphinidin is a dominant anthocyanidin component in red wine and berries, known to have effective biological functions in the prevention of oxidative stress, angiogenesis, inflammation and metastasis, as well as carcinogenesis [17,18,19]. An increasing number of studies have identified the potential protective effects of D against carcinogenesis of the breast [20], prostate [21], lungs [22], liver [23] and colon [24] and against fibrosarcoma [25]. Nevertheless, little is known about its effects on epithelial OC, a disease that is associated with a low survival rate, poor prognosis and a high rate of recurrence. Lim and Song [26] demonstrated evidence, using the SKOV3 human OC cell line, that D is an inhibitor of epithelial OC cell proliferation through inactivating the phosphorylation of AKT, P70S6K, S6 and ERK1/2 proteins in the PI3K/AKT and ERK1/2 MAPK signaling pathways and that these cell-signaling pathways may be a pivotal therapeutic target for the prevention and therapy of epithelial OC, including paclitaxel-resistant OC. It was found that D increased the rate of apoptosis via the induction of DNA fragmentation in SKOV3 cells [26].

We found that D was the most cytotoxic compound for OC cells among the flavonoids studied by us. There are no reports so far on the inhibition of glycolysis by D. Therefore, the most probable mechanisms of the anticancer action of D do not involve glycolysis, and a combination of D with 3-BP could attack simultaneously two independent cellular targets, creating the possibility of a synergistic interaction. The aim of this study was to examine how 3-BP and D, as well as 3-BP in combination with D, affect the survival and mobility, as well as biochemical parameters such as the glutathione (GSH) and ATP content and the level of reactive oxygen species (ROS), in two human OC cell lines of differ origin (SKOV3 and PEO1) and human lung fibroblast (MRC-5) serving as a normal (noncancerous) cell line. PEO1 is a high-grade serous, and SKOV3 a non-serous, OC cell line [27,28]. We expect that this combined approach should be more deleterious to OC cells than attacking only one target inside the cells.

## 2. Results

### 2.1. Delphinidin Chloride and 3-Bromopyruvic Acid Cytotoxicity

**Figure 2:**
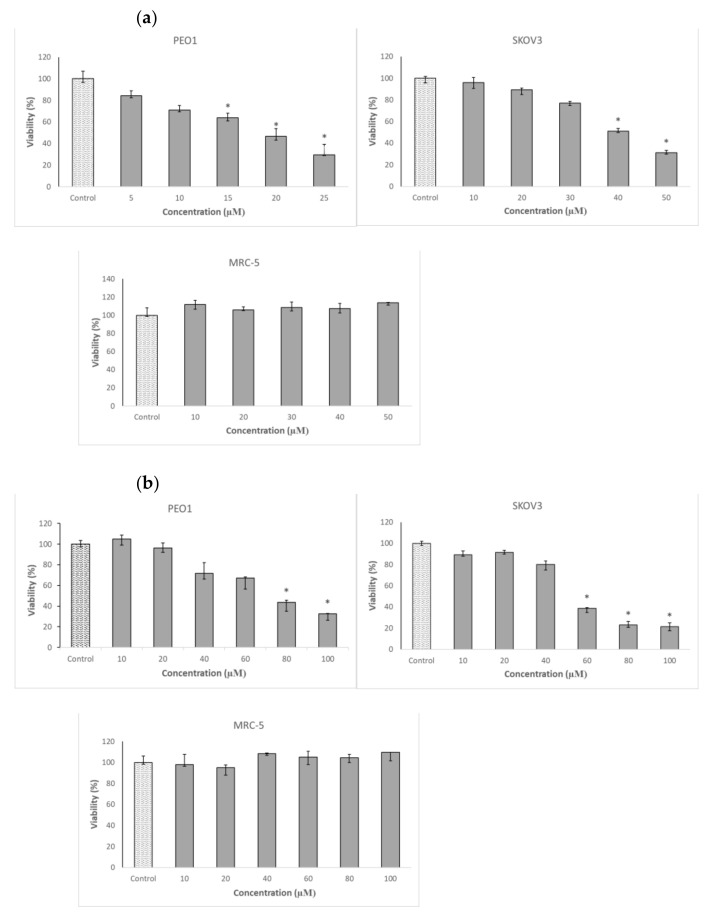
Effect of 3-bromopyruvic acid (3-BP) (**a**) and delphinidin (D) (**b**) on the survival of PEO1, SKOV3 and MRC-5 cells estimated by the Neutral Red assay. The cells were treated with D in the concentration range of 10–100 μM and 3-BP at concentrations of 5–25 μM for PEO1 and 10–50 μM for MRC-5, as well as SKOV3. Nontreated cell were used as a control. * *p* < 0.05 with respect to the control (Kruskal-Wallis test; *n* ≥ 6).

**Figure 3:**
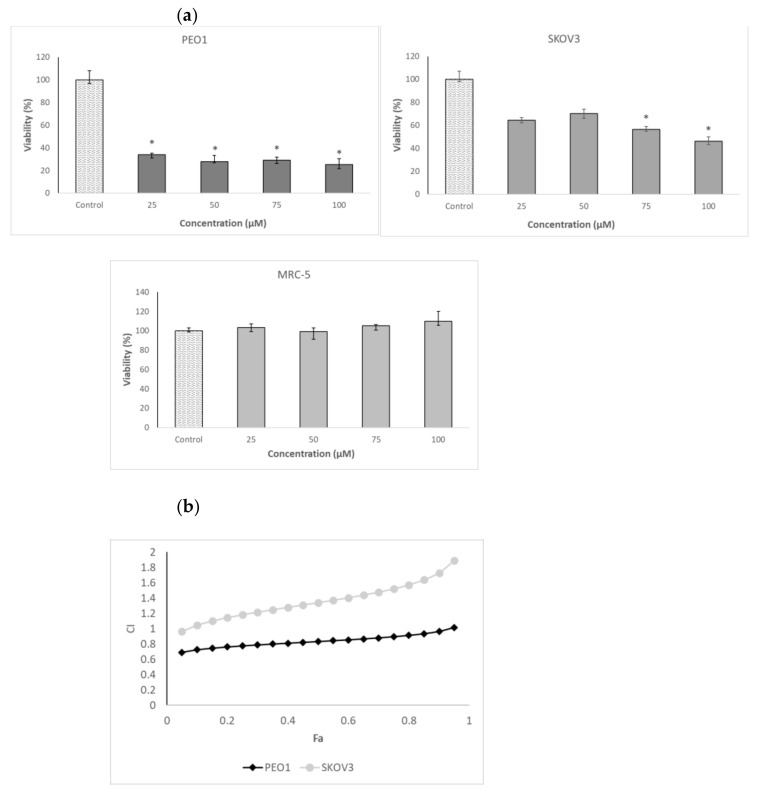
Effect of the combined action of 3-BP and D on the viability of the PEO1, SKOV3 and MRC-5 cells. Survival of the cells treated with 3-BP at a concentration corresponding to ~half maximal inhibitory concentration (IC_50_) (SKOV3 and MRC-5: 40 μM and PEO1: 20 μM) and various concentrations of D indicated on the axis of the abscissae (**a**). Plot of the combination index (CI) as a function of the effect level (Fa) for the combined action of 3-BP and D. Concentrations of 3-BP and D: 20, 40, 60 and 80 μM (**b**). * *p* < 0.05 with respect to the control (Kruskal-Wallis test; *n* ≥ 6).

Cancer ovary cells of both lines studied, especially PEO1, showed a high sensitivity to 3-BP. In contrast, the survival of MRC-5 fibroblasts, used as a control cell line, was not compromised by 3-BP up to a concentration of 50 µM (Table 1 and Figure 2a). The cells were not especially vulnerable to several flavonoids studied, including four anthocyanidins, in the vast majority of cases, the half maximal inhibitory concentration (IC_50_) values being higher or much higher than 100 µM. The only exception was D, with IC_50_ values below 100 µM for both PEO1 and SKOV3 cells. Delphinidin did not affect the survival of MRC-5 fibroblasts in the concentration range studied (up to 100 µM) (Table 1 and Figure 2b). When cells were treated with D together with 3-BP employed at a concentration corresponding to the IC_50_ for a given cell line, D decreased further the survival of PEO1 cells, the decrease being weakly dependent of the D concentration in the concentration range of 25–100 µM. The survival of 3-BP treated SKOV3 cells were not decreased by D but even showed a tendency for increasing in the presence of 25-µM and 50-µM D but were compromised by 100-µM D. The survival of MRC-5 cells was not affected by the combined action of 3-BP and D in the concentration range applied (Figure 3a).

### 2.2. Mode of Interaction of 3-BP and Delphinidin

When the interaction of 3-BP and D were analyzed in the formalism of Chou and Tatalay [29,30], a plot of the combination index (CI) for the combined action of both compounds as a function of their cytotoxicity demonstrated a mostly synergistic interaction in the case of PEO1 cells (CI <1) and additive (CI ≈ 1) or antagonistic (CI >1) in the case of SKOV3 cells (Figure 3b).

### 2.3. ATP Level and GSH Content 

**Figure 4:**
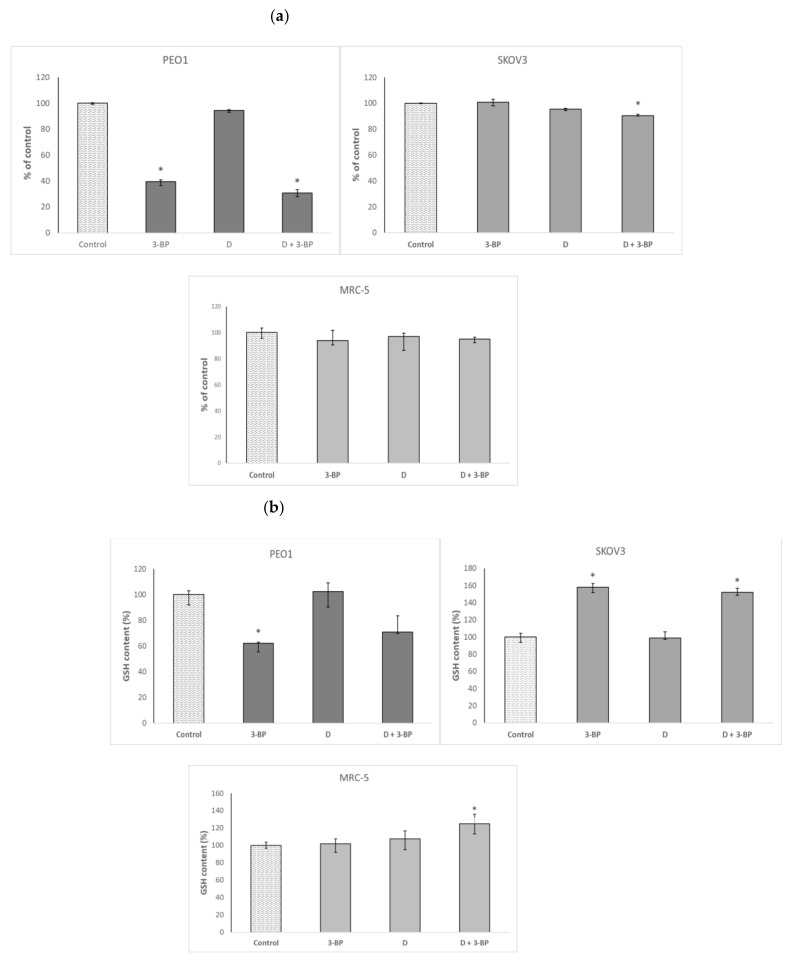
Effect of 3-BP and delphinidin (D) on the ATP (**a**) and GSH content (**b**) of PEO1, as well as SKOV3 and MRC-5 cells. *p < 0.05 with respect to the control (Kruskal-Wallis test; *n* ≥ 6).

3-BP induced a decrease in the ATP content of the PEO1 cells. Delphinidin alone did not change the ATP content significantly, but the combined action of 3-BP and D decreased it even somewhat more than 3-BP alone. In SKOV3 cells, neither 3-BP nor D affected the ATP content, but the combined action of both compounds induced a decrease of this parameter. The ATP content of the MRC-5 fibroblasts was not affected by 3-BP, D or a combination of both (Figure 4a). 

3-BP decreased the GSH level in PEO1 cells. Delphinidin did not affect the GSH level, and the combined action of both compounds lowered it similarly to 3-BP alone, though this decrease was devoid of statistical significance due to the scattering of data. In SKOV3 cells, 3-BP and the combined action of 3-BP+D increased the level of GSH, while D alone was without effect. In the case of MRC-5 fibroblasts, neither 3-BP or D alone modified the GSH level, but a combined action of both compounds increased it (Figure 4b).

### 2.4. The Level of Reactive Oxygen Species

**Figure 5:**
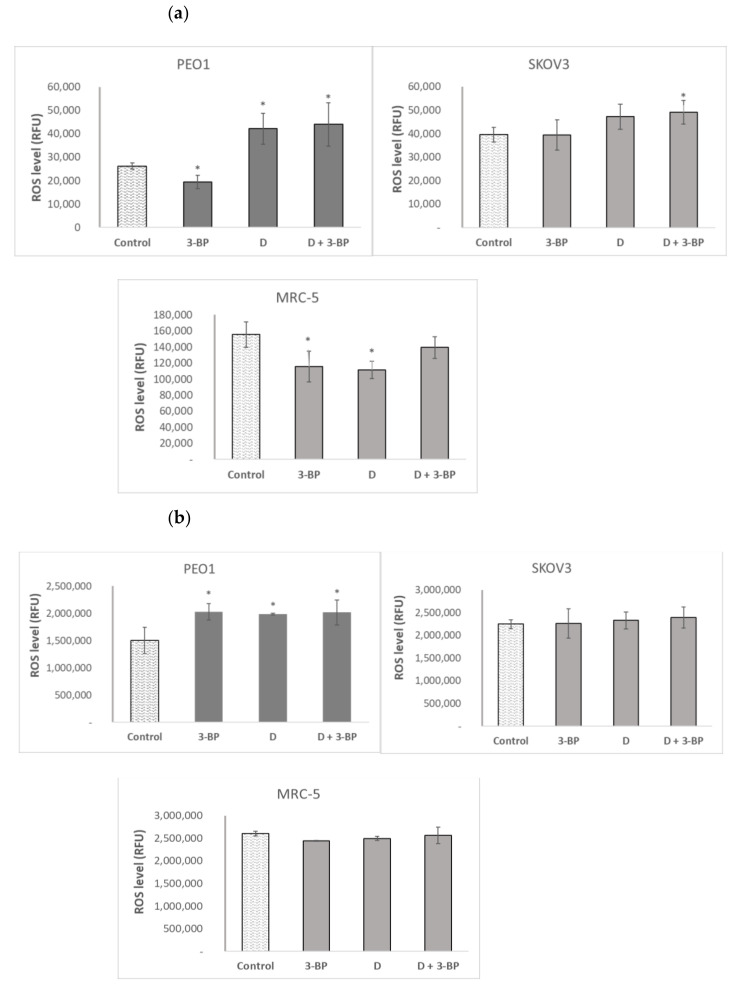
Effect of 3-BP and delphinidin (D) on the level of reactive oxygen species (ROS) estimated with dihydroethidium (DHE, (**a**) and 2’,7’-dihydrodichlorofluorescein diacetate (DCFDA, (**b**). * *p* < 0.05 with respect to the control (one-way ANOVA and Least Significant Difference (LSD) post-hoc test; *n* ≥ 3).

3-BP decreased the level of reactive oxygen species (ROS) detectable with DHE (mainly superoxide) in PEO1 and MRC-5 cells, not affecting it in SKOV3 cells. Delphinidin increased the level of DHE-detectable ROS in PEO1 cells and decreased it in MRC-5 fibroblasts. The combined action of 3-BP and D increased the level of DHE-detectable ROS in cancer cells of both lines, not affecting it in the fibroblasts (Figure 5a).

2’,7’-Dichlorofluorescein (DCFDA), reacting with a broader range of ROS than DHE, showed a different response pattern. The level of DCFDA-reactive ROS was increased by 3-BP, D and 3-BP+D in PEO1 cells and was not affected by these compounds in the SKOV3 and MRC-5 cells (Figure 5b). 

### 2.5. Mitochondrial Potential and Mitochondrial Mass

**Figure 6:**
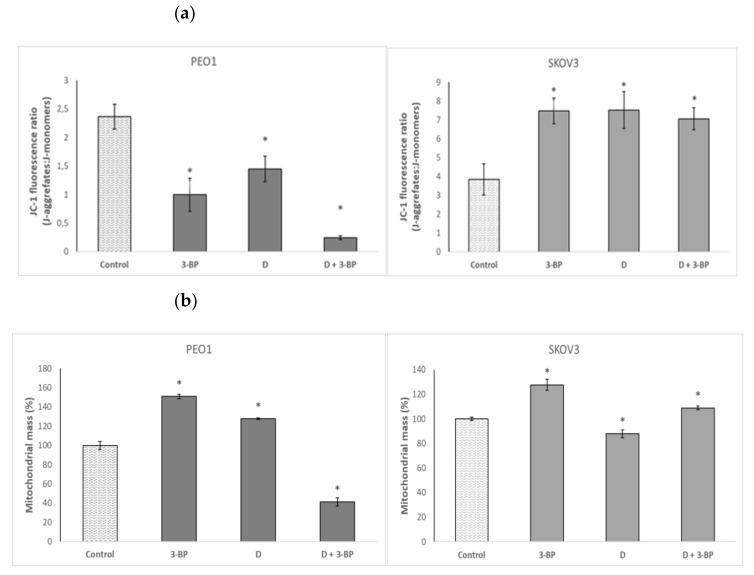
Effect of 3-BP and delphinidin (D) on mitochondrial staining with the JC-1 probe (**a**) and mitochondrial mass (**b**) of PEO1 and SCOV3 cells. * *p* < 0.05 with respect to the control (one-way ANOVA and LSD post-hoc test; *n* ≥ 3).

Both 3-BP and D decreased the mitochondrial potential in PEO1 cells, as demonstrated by a decrease in the ratio of red to green fluorescence intensity of the 5,5′,6,6′-tetrachloro-1,1′,3,3′-tetraethyl-imidacarbocyanine iodide, 5,5′,6,6′-tetrachloro-1,1′,3,3′-tetraethylbenzimidazolocarbocyanine iodide (JC-1) probe. The combined action of 3-BP and D induced a dramatic decrease of the mitochondrial potential in PEO1 cells. In contrast, the mitochondrial potential was increased by 3-BP, D and the combination of 3-BP and D in SKOV3 cells (Figure 6a). The mitochondrial mass was increased by 3-BP and D but strongly decreased by the combined action of both compounds in PEO1 cells. In SKOV3 cells, D decreased while 3-BP and 3-BP in combination with D increased the mitochondrial mass (Figure 6b). 

Cultures of PEO1 and SKOV3 cells treated as described in Section 4.5 were stained with Atto-488-phalloidin (Figure 6a) and Mitotracker Deep Red FM (Figure 6b), which targeted the cytoskeletal actin filaments and the intracellular mitochondrial network, respectively. In PEO1 cells, 3-BP induced about a 51% increase in the intensity of staining with Atto-488-phalloidin, D caused about a 17% decrease and 3-BP plus D induced about a 26% decrease in the signal intensity with respect to the control. In SKOV3 cells, the intensity of staining with Atto-488-phalloidin decreased by about 33%, 67% and 38% after treatment with 3-BP, D and 3-BP plus D, respectively (Figure 6a). The intensity of staining of PEO1 cells with Mitotracker Red decreased by about 7% after treatment with 3-BP, increased by about 7% after treatment with D and decreased by about 41% after treatment with 3-BP and D. The intensity of staining of SKOV3 cells with Mitotracker Red decreased by about 39%, 94% and 52% after treatment with 3-BP, D and 3-BP plus D, respectively, with respect to the control (Figure 6b).

### 2.6. Atto-488-Phalloidin and Mitotracker Labeling 

**Figure 7:**
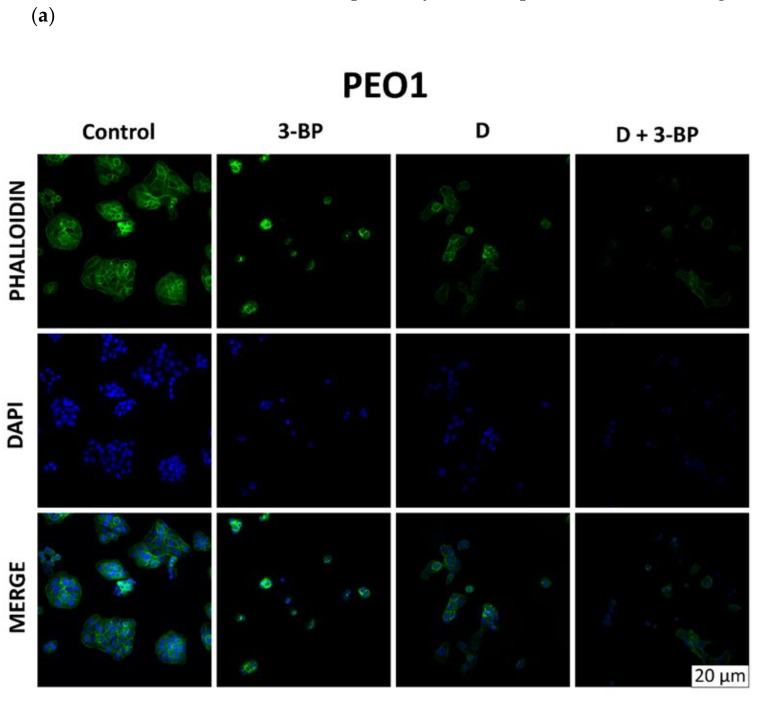

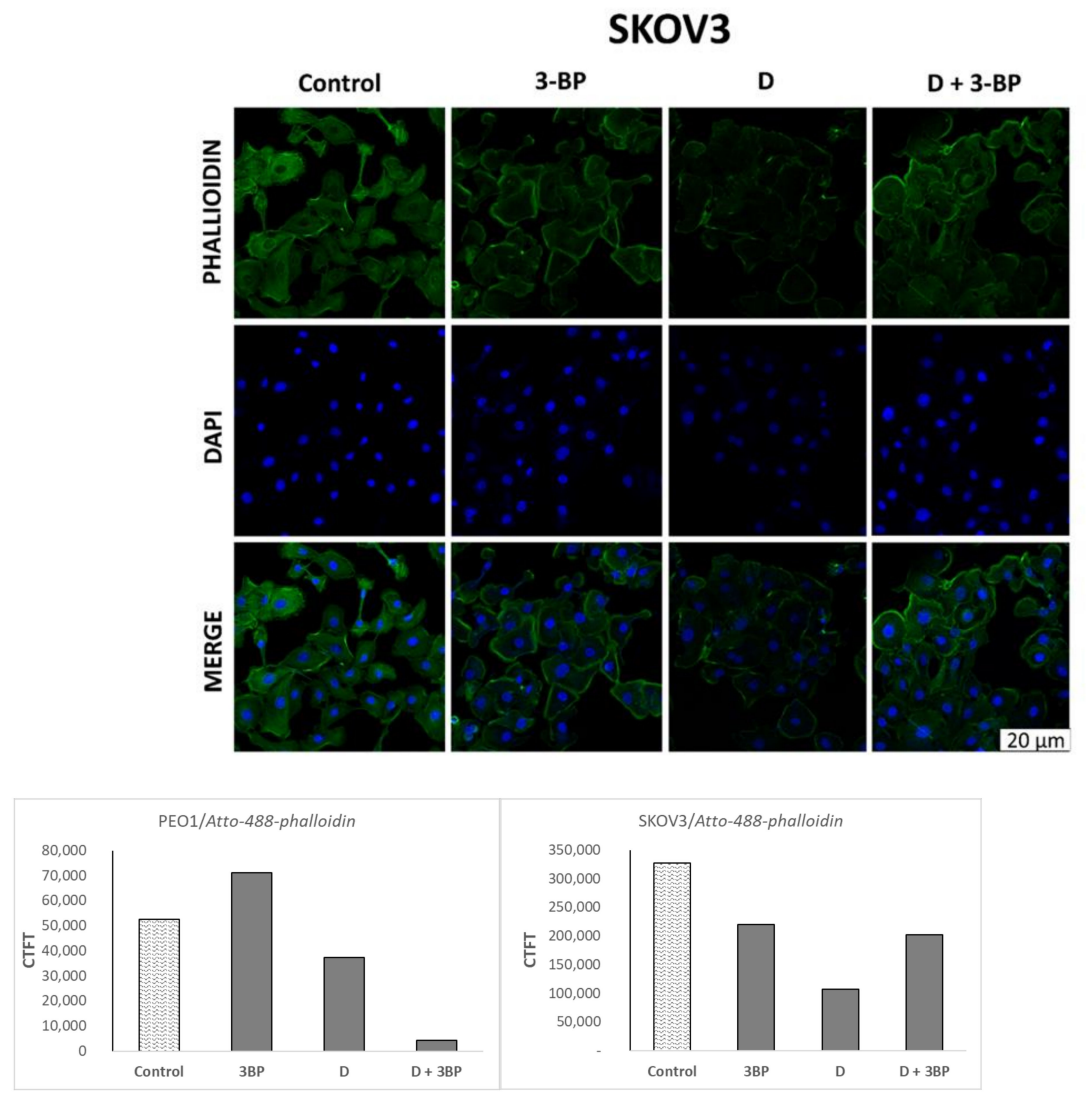

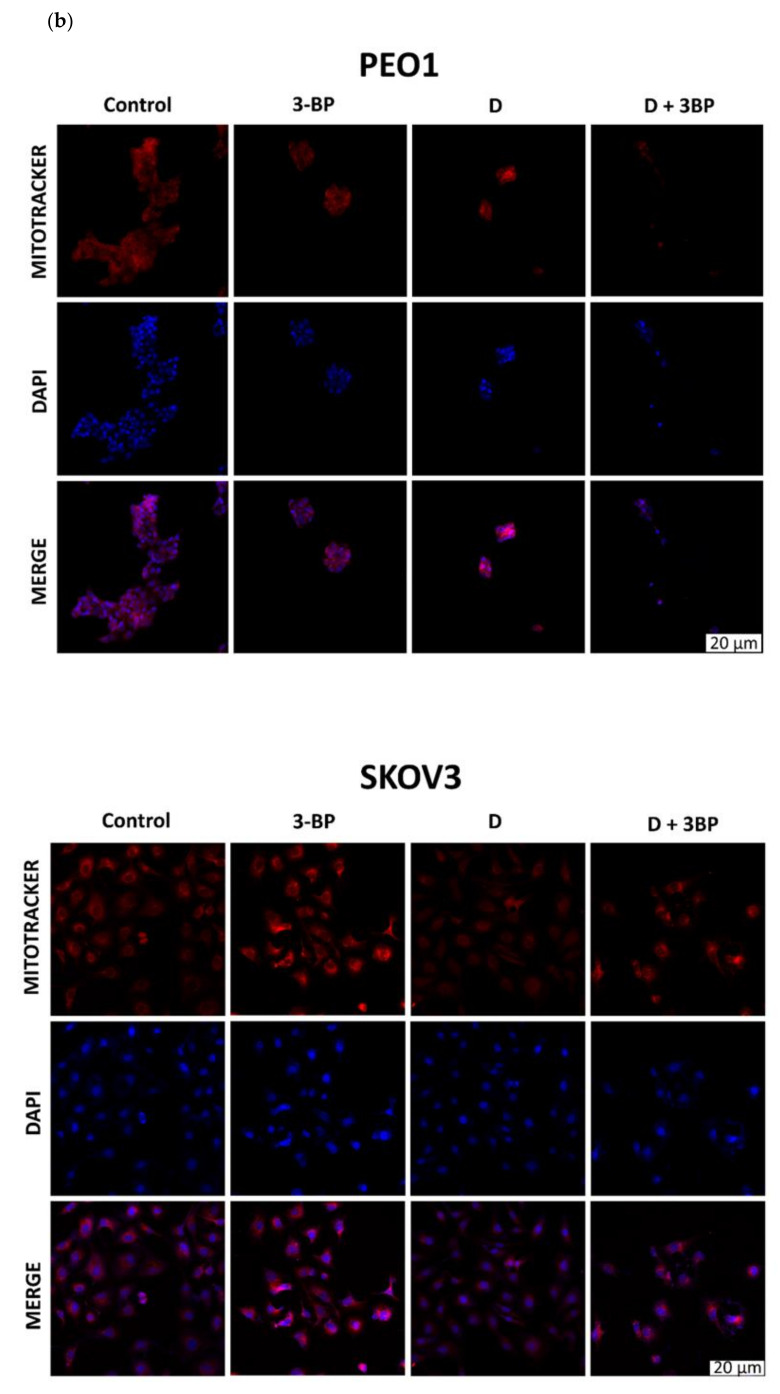

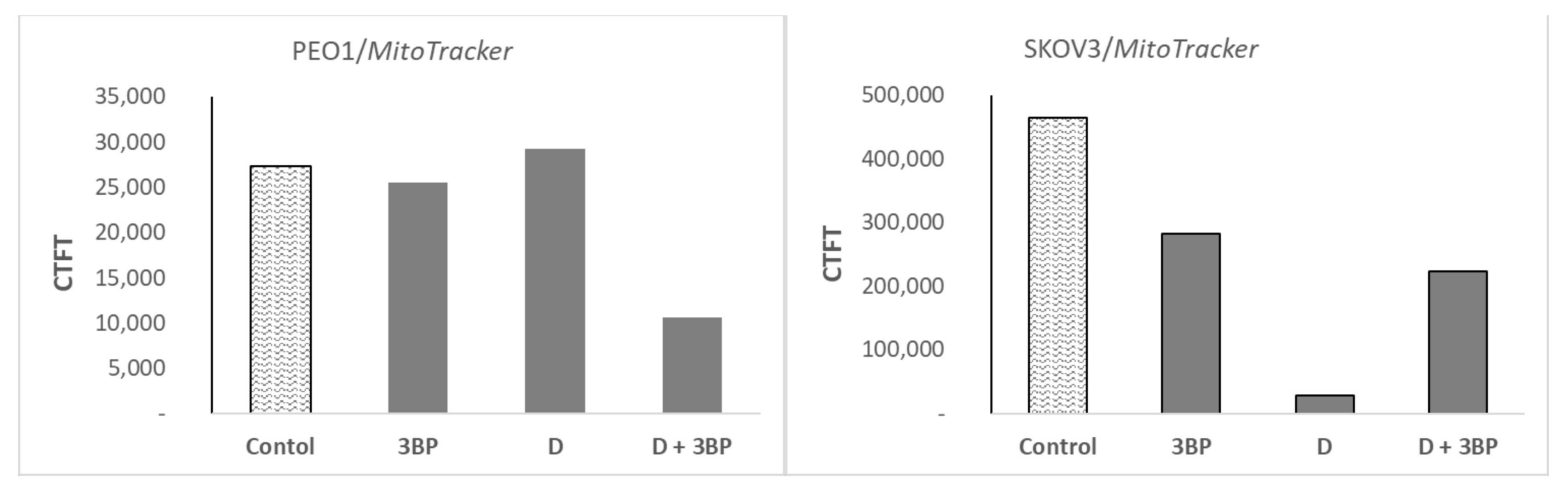
Morphological changes in PEO1 and SKOV3 cells (confocal images, 65×). Effect of 3-BP, D and D plus 3-BP on the staining of PEO1 and SKOV3 cells with phalloidin (**a**) and Mitotracker Red (**b**).

Cultures of PEO1 and SKOV3 cells were stained with and Atto-488-phalloidin (Figure 7a) and Mitotracker Deep Red FM (Figure 7b), which target the cytoskeletal actin filaments and the intracellular mitochondrial network, respectively. In PEO1 cells, 3-BP induced about a 51% increase in the intensity of staining with Atto-488-phalloidin, D caused about a 17% decrease and 3-BP plus D induced about a 26% decrease in the signal intensity with respect to the control. In SKOV3 cells, the intensity of staining with Atto-488-phalloidin decreased by about 33%, 67% and 38% after treatment with 3-BP, D and 3-BP plus D, respectively (Figure 7a). The intensity of staining of PEO1 cells with Mitotracker Red decreased by about 7% after treatment with 3-BP, increased by about 7% after treatment with D and decreased by about 41% after treatment with 3-BP and D. The intensity of staining of SKOV3 cells with Mitotracker Red decreased by about 39%, 94% and 52% after treatment with 3-BP, D and 3-BP+D, respectively, with respect to the control (Figure 7b). 

### 2.7. Cell Migration

**Figure 8:**
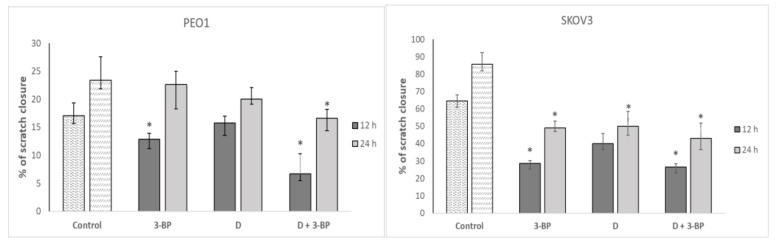
Effect of 3-BP and delphinidin (D) on the migration of PEO1 and SCOV3 cells. * *p* < 0.05 with respect to the control (Kruskal-Wallis test; *n* = 12).

The migration of PEO1 cells was significantly inhibited by 3-BP and the combination of 3-BP and D, while the migration of SKOV3 was decreased by 3-BP, D and 3-BP plus D (Figure 8). 

### 2.8. Cell Apoptosis and Necrosis

**Figure 9:**
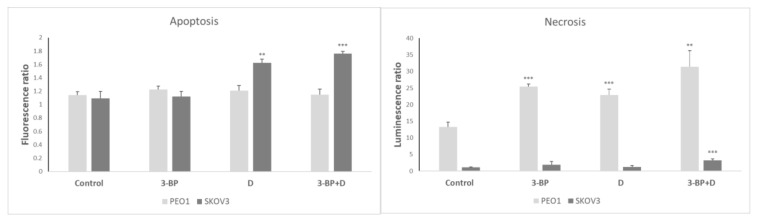
The induction of apoptosis and necrosis by 3-BP, D and 3-BP+D in PEO1 and SKOV3 cells (after 24 h). Fluorescence and luminescence, respectively, of control cells at 0 time assumed as 1. ** *p* < 0.01 and *** *p* < 0.001 with respect to the control (one-way ANOVA and LSD post-hoc test; *n* = 3).

The evaluation of the induction of apoptosis and necrosis in PEO1 and SKOV3 cells by 3-BP, D and both compounds together showed that D and the combined action of 3-BP and D induced apoptosis in SKOV3 but not in PEO1 cells, while 3-BP, D and 3-BP+D provoked a considerable induction of necrosis of PEO1 cells. The combined action of 3-BP and D induced a much lower induction of necrosis in SKOV3 cells (Figure 9).

## 3. Discussion

3-Bromopyruvic acid, a glycolytic inhibitor and alkylating agent, has been proposed as an anticancer agent, and its efficacy has been demonstrated at the cellular level, as well as in animal experiments [11,31,32]. Cells of four OC cell lines were found to have IC_50_ values for 3-BP in the range of 16–84 µM [13]. The IC_50_ values obtained in this study for two cell lines, not covered by the previous study, are contained within this range. 

Delphinidin is one of six most commonly occurring anthocyanidins, characterized by the highest number of hydroxyl groups in the molecule, which underlies its high antioxidant and perhaps other activities. Delphinidin was found to inhibit proliferation and induce apoptosis in many different cancer models, including colon, uterine, breast and prostate, but the sensitivity of cancer cells to D showed considerable differences, depending on cell line and experimental conditions applied in different laboratories. In prostate cancer cells, D did not affect the viability of DU145 and PC3 cell lines at concentrations of up to 150 µM, but concentration-dependently decreased the viability of LnCaP cells starting from a concentration of 50 µM [33]. Among six types of anthocyanidins, D was reported to have the strongest antitumorigenic activity with the inhibition of cell proliferation and migration [34]. Delphinidin, at concentrations of 100–200 µM, decreased the viability of NHF, Caco-2 and HeLa cells [35]. D (50–200 µM) decreased, concentration-dependently, the viability of MCF-7 but did not affect significantly the viability of AGS, SF-268, HCT-116 and NCI H460 cells [36]. In another study, the IC_50_ values of D for A549, NCI-H441 and SK-MES-1 cells were reported to be 55, 58 and 44 μM, respectively [22]. The IC_50_ of D for HCT116 cells was found to be 110 μM [24] and 38 μM for LoVo cells [37]. In other studies, the IC_50_ of D was found to be 90 μM for 22Rν1 cells [38] and 35 μM for HT29 cells [39]. One factor contributing to this diversity of results may consist of the use of viability tests based on the use of tetrazolium salts in some papers. Redox active flavonoids—among them, anthocyanidins—may directly reduce tetrazolium salts, leading to artefactual results [40]. Therefore, we assayed the cellular viability using Neutral Red, which is not redox-active. 

D was also demonstrated to inhibit glyoxalase I, the rate-limiting enzyme for the detoxification of methylglyoxal, a side-product of glycolysis, which is able to induce apoptosis. D is a much better inhibitor of this enzyme than other anthocyanidins studied, with an IC_50_ of 1.9 µM [41], and this effect may contribute to the cytotoxicity of D.

PEO1 cells were more sensitive to 3-BP than SKOV3 cells. 3-BP depletes the cells of ATP, which is a consequence of the inhibition of glycolysis and, to a lesser degree, impairment of mitochondrial ATP production by this compound. Energy depletion seems to be the main factor of 3-BP cytotoxicity [42,43,44,45]. The difference in sensitivity to 3-BP between the cell lines studied may be related to the greater dependence of energy metabolism of PEO1 cells on glycolysis with respect to SKOV3 cells (> 75% vs. < 25%, respectively [46], which should lead to a higher sensitivity of PEO1 cells to glycolytic inhibitors. This difference between both cell lines can also contribute to the greater effect of 3-BP and 3-BP+D on the ATP level in PEO1 cells as compared with SKOV3 cells (Figure 4a).

Our results confirm the high sensitivity of OC cells to D. In our hands, the IC_50_ of SKOV3 cells for D was 40.5 μM, and PEO1 cells were even more sensitive to D (IC_50_ of 18.7 μM; Table 1). These concentrations are still too high to suggest the use of D as a candidate for single-compound therapeutic application in the therapy of ovarian cancer. However, D can be very promising in potentiating the action of 3-BP on OC cells, but its efficacy depends on the cell type. In PEO1 cells, 25–100-μM D in combination with 3-BP applied at a concentration corresponding to the IC_50_ considerably reduced the viability. In contrast, in SKOV3 cells, the potentiation of the effect of 3-BP required higher D concentrations, while lower D concentrations even increased the viability with respect to the treatment with 3-BP alone (Figure 3a), in agreement with the antagonistic effect indicated by the Chou-Tatalay plot (Figure 3b). 

The decrease in the viability of cells treated with 3-BP and D is contributed by cell death, which is necrotic in PEO1 cells and apoptotic in SKOV3 treated with D and 3-BP+D, with the contribution of necrosis in SKOV3 cells subjected to the action of 3-BP+D (Figure 9).

Interestingly, both 3-BP and D were reported to be effective against cancer cells resistant to classical chemotherapy. Multidrug-resistant SKOV3 cells, which were several times less sensitive than not multi-resistant SKOV3 cells to cisplatin, carboplatin, paclitaxel and 5-fluorouracil, showed similar sensitivity to 3-BP (IC_50_ of 22 ± 4 μM vs. 19 ± 3 μM) [47]. MDCKII cells overexpressing the ABCB1 multidrug transporter were more sensitive to 3-BP than the parent cell line [42]. 3-BP significantly decreased the IC_50_ values of doxorubicin, paclitaxel, daunorubicin and epirubicin in MCF-7 cells resistant to adriamycin, whereas it did not alter the IC_50_ values in parent MCF-7 cells [48]. Delphinidin was also reported to be effective against multidrug-resistant cells. The LoVo cell subline resistant to adriamycin was more sensitive to D than the parent cell line (IC_50_ of 16 ± 2 μM vs. 38 ± 3 μM) [37].

In order to get an insight into the mechanisms of action of 3-BP and D on the OC cells, we studied the effect of these compounds on the redox status of the cells, analyzing the content of GSH and the level of ROS. Our results point to a considerable difference in the response of the GSH content of both cell lines studies to 3-BP. While D did not affect the GSH content in the cells of both lines, 3-BP alone and in combination with D decreased the GSH content in PEO1 cells and increased it in SKOV-3 cells (Figure 4b). Glutathione depletion, due to the formation of 3-pyruvylglutathione conjugate [49], is another important factor in the cellular action of 3-BP [49,50,51,52]. The reaction of PEO1 cells is typical in this respect. The increase of the GSH level in SKOV3 cells seems to be a manifestation of an overcompensative stimulation of GSH synthesis in response to an initial GSH depletion. Such a reaction has been demonstrated to occur in SH-SY5Y cells treated with another GSH-depleting agent, 6-hydoxydopamine [53,54]. Such a reaction may contribute to the greater resistance of SKOV3 cells to 3-BP as well. A greater decrease of the GSH level of PEO1 cells in comparison to SKOV3 cells was also noted in response to other agents (Heregulin-β1 in combination with lapatinib, erlotinib or bexarotene) [55].

3-BP was found to increase the cellular level of ROS in various cell types [56,57,58,59]. Our results support this view with respect to PEO1 cells (ROS assay with DCFDA; Figure 5b) but not to SKOV3 cells (Figure 5a,b). We found also a stimulation of ROS formation by D and 3-BP+D, especially in PEO1 cells, indicating that D behaves as a prooxidant rather than antioxidant, in this case (Figure 5a,b).

Delphinidin was reported to also decrease the GSH level and induce ROS formation in other cancer cells. In LoVo cells, D increased the level of ROS and depleted the cellular GSH [37]. Delphinidin-rich anthocyanin extracts of *Hibiscus sabdariffa* L. decreased the mitochondrial potential and increased the level of DCFDA-detectable ROS in MCF-7 cells [60].

The decrease of the mitochondrial potential induced by 3-BP, D and, especially, 3-BP plus D in PEO1 cells (Figure 6a) contrasted with its increase in SKOV3 cells, and the decrease in the mitochondrial mass induced by 3-BP plus D in PEO1 but not in SKOV3 cells (Figure 6b) may be a factor determining the synergistic action of both compounds in PEO1 but not in SKOV3 cells (Figure 3b). Perhaps the differences in the response of the cell lines studied to 3-BP and D reflects the behavior of high-grade serous (PEO1) and non-serous OC cell lines, but verification of this hypothesis would require further studies. 

Delphinidin was reported to reduce the brain-derived neurotrophic factor (BDNF)-induced migration of SKOV3 cells. These effects were ascribed to a blockade of the PI3K/AKT and ERK1/2 MAPK pathways [61,62]. Our results confirm the effect of 3-BP [12] and D [61,62] on the cellular mobility, showing that D potentiates the effect of 3-BP (Figure 8). In both cell lines, D alone and in combination with 3-BP decreased the staining with phalloidin (Figure 7a). The decreased content/disorganization of actin filaments may explain the decreased cell mobility.

Delphinidin is unstable in cell culture media, being not detectable in Dulbecco’s modified Eagle’s medium (DMEM) medium after 30 min, and was most sensitive to degradation from among the five anthocyanidins studied. Gallic acid, 4-hydroxybenzoic acid, protocatechuic acid, 4-hydroxybenzoic acid, vanillic acid and syringic acid were the main decomposition products. However, the IC_50_ values of the decomposition products were much higher than that of D, with the exception of gallic acid, which was also unstable, so it is not probable that the toxicity of D is mediated by these decomposition products. The destruction of D generates also hydrogen peroxide, but 75-µM D produced about 10-µM H_2_O_2_ [39], which is not probable to affect cell survival significantly. In our opinion, D is rapidly taken up by the cells, where it is protected from oxidative degradation by reducing the environment of the cell interior and seems to be responsible itself for the cytotoxic effects.

## 4. Materials and Methods 

### 4.1. Reagents and Materials

#### 4.1.1. Cells 

The human OC cell line (SKOV3 (HTB-77)) and human lung normal fibroblast cell line (MRC-5 (CCL-171)) were obtained from the American Type Culture Collection (ATCC). We also used the second OC cell line derived from humans (PEO1 (10032308)) purchased in the European Collection of Authenticated Cell Cultures (ECACC). The SKOV3 cell line was a hypodiploid OC derived from a 64-year-old Caucasian female with an ovarian serous cyst adenocarcinoma. The number of chromosomes was 43, occurring in over 60% of the cells. These adenocarcinoma cells were positive for many antigens generally used to identify epithelial cancer—for example: EMA (epithelial membrane antigen), VIM (vimentin) and cytokeratin alike.

The second OC cell line used in the experiments was PEO1 derived from a malignant effusion from the peritoneal ascites of a patient with a poorly differentiated serous adenocarcinoma after treatment with chlorambucil, 5-fluorouracil and cisplatin. This cell line was positive for hormone receptors like the estrogen receptor.

Normal human fibroblasts were used as control cells. The MRC-5 fibroblast line was derived from normal lung tissue of a 14-week-old male fetus. This is a normal diploid human cell line with 46 XY karyotypes.

#### 4.1.2. Disposables for Cell Culture

Flasks (75 cm^2^; cat. no. 156499), 8-well chamber slide w/ removable wells (cat.no. 154534) and 96 -well white plate (cat. no. 165306) were provided by Thermo Fisher Scientific (Waltham, MA, USA). Transparent 96-well culture plates (cat. no 655180), black flat-bottom 96-well plates (cat. no. 655209) and 24-well plates (cat. no. 662160) were obtained from Greiner (Kremsmünster, Austria). Other sterile cell culture materials were provided by Nerbe (Winsen, Germany). 

#### 4.1.3. Cell Culture Media

Cell culture medium (McCoy’s 5A (cat. no 22330-021), Roswell Park Memorial Institute (RPMI) medium + GlutaMAX (cat. no 72400-021), Dulbecco’s Modified Eagle Medium) (DMEM) + GlutaMAX (cat. no. 21885-025)) and Dulbecco’s phosphate-buffered saline (DPBS) (cat. no. 14040-117) were purchased from Thermo Fisher Scientific (Waltham, MA, USA). 

#### 4.1.4. Other Reagents

Fetal bovine serum (FBS; cat. no. S1813), penicillin–streptomycin solution (cat. no. L0022), trypsin-EDTA solution (10x) (cat. no. X0930) and phosphate-buffered saline without Ca^2+^ and Mg^2+^ (cat. no. P0750) were obtained from Biowest (Nuaillé, France). 0.33% Neutral Red solution (NR) (cat. no. N2889), 0.4% trypan blue solution (cat. no. T8154), N-ethylmaleimide (NEM) (cat. no. E3876), trichloroacetic acid (TCA) (cat. no. T4885), diethylenetriamine-pentaacetic acid (DTPA) (cat. no. D1133), L-ascorbic acid (cat. no. A0278), 2’,7’-dichlorofluorescein (DCFDA) (cat. no. 35845), dihydroethidium (DHE) (cat. no. 37291), dimethyl sulfoxide (DMSO) (cat. no. D2438), *o*-phtaldialdehyde (OPA) (cat. no. P1378), bromopyruvic acid (cat. no. 16490), phalloidin-atto 488 (cat. no. 49409) and Triton X-100 (cat. no. 9002-93-1) were provided by Sigma-Aldrich (St. Louis, MO, USA). Ethanol (96%; cat. no. 396420113) and glacial acetic acid (cat. no. 568760114), as well as methanol (cat. no. 6219900110) were obtained from Avantor Performance Materials, (Gliwice, Poland). Formaldehyde solution (37%; cat. no. 114321734) was provided by Chempur (Piekary Śląskie, Poland). Delphinidin chloride (cat. no 528-53-0) was purchased in EXTRASYNTESE (Genay, France) Mitotracker Deep Red FM (cat. no. M22426) was purchased from Thermo Fisher Scientific (Waltham, MA, USA). CellTiter-Glo® Luminescent Cell Viability Assay (cat. no. G7571) was obtained from Promega (Madison, WI, USA). JC-1 Mitochondrial Membrane Potential Assay Kit was purchased from Abnova (Taiwan, China).

Stock solutions of 3-BP and delphinidin chloride were freshly prepared in DMSO and then diluted in a proper cell culture medium. Absorptiometric fluorometric and luminescence measurements were done in a Spark multimode microplate reader (Tecan Group LTD., Männedorf, Switzerland). Transmission light microscope observations were done in an inverted Olympus CKX53 microscope (OLYMPUS, Tokyo, Japan). Changes in mitochondrial and cytoskeleton were observed using an inverted confocal microscope ZEISS LSM 710 (Oberkochen, Germany). Cell migration was documented using an Olympus CKX53 microscope with a U-TV0.5XC-3 digital microscope camera (OLYMPUS, Tokyo, Japan). 

### 4.2. Cell Culture

SKOV3 cells were cultured in McCoy’s 5A medium, PEO1 cells were cultured in RPMI + GlutaMAX and MRC-5 cells were cultured in DMEM + GlutaMAX. Media used in the experiments were supplemented with 1% v/v penicillin/streptomycin solution and 10% heat inactivated fetal bovine serum (FBS). Cells were incubated at 37 °C under 5% carbon dioxide and 95 °C humidity. Cells were passaged at about 85% confluence. Cell viability was estimated by the trypan blue exclusion test. Cells were counted in a Thoma hemocytometer (Superior Marienfeld, Lauda-Königshofen, Germany).

### 4.3. Delphinidin Chloride and 3-Bromopyruvic Acid Cytotoxicity

SKOV3 and PEO1 cells were seeded in a clear 96-well plate at a density of 1 × 10^4^ cells/well in 100-µL culture medium. MRC-5 cells were seeded at a density of 7.5 × 10^3^/well and allowed to attach for 24 h at 37 °C. After incubation, the cells were treated with delphinidin chloride in a concentration range of 10–100 µM and 3-BP at concentrations between 5–25 µM for PEO1 and 10–50 µM for MRC-5 and SKOV3. Nontreated cells were used as a control. Stock solutions of tested substances were prepared in DMSO and their working solutions in culture media. The DMSO concentration was adjusted to 0.2% in all samples, which had no significant effect on the treated cells. After 24-h exposure time, the medium was removed and replaced with 100 µL of 2% neutral red solution, and the cells were incubated at 37 °C for 1 h. Then, the cells were washed with PBS; fixed with 100 µL/well of 50% ethanol, 49% H_2_O and 1% glacial acetic acid and shaken (700 rpm) at room temperature for 20 min. Absorbance was measured at 540 nm against 620 nm.

### 4.4. Mode of Interaction of 3-BP and Delphinidin

Ovarian carcinoma cells were seeded at a density of 1 × 10^4^ cells/well in a 96-well transparent plate and incubated for 24 h to allow for maximal adherence. Subsequently, the cells were treated with D, 3-BP and both factors simultaneously at concentrations of 20, 40, 60 and 80 µM. After 24-h exposure, the cytotoxicity was determined with neutral red as described above. The mode of interaction of both compounds was analyzed according to the model of Chou and Tatalay [29,30] using CompuSyn software (2005; https://www.combosyn.com/register.html).

### 4.5. ATP Level

To determine the ATP level in the cells, the CellTiter-Glo® Luminescent Cell Viability Assay (Promega) was used. This method is based on the ATP-dependent conversion of luciferin to the luminescent oxyluciferin. Briefly, cells were seeded in a white/clear bottom 96-well plate with a density of 1 × 10^4^/well (SKOV3 and PEO1) or 7.5 × 10^3^/well (MRC-5). After 24-h incubation, the cells were treated with 25-µM D, a IC_50_ concentration of 3-BP (SKOV3 and MRC-5: 40 µM and PEO1: 20 µM) and both compounds simultaneously. The ATP level was determined after 24-h exposure to the tested compounds. The plate was equilibrated at room temperature for approximately 25 minutes. Next, 100 µL of the CellTiter-Glo® Reagent was added to each well, and the plate was shaken for 2 minutes. Then, the plate was left at room temperature for 10 minutes, and the luminescence was measured in a Spark multimode microplate reader (Tecan Group LTD., Männedorf, Switzerland).

### 4.6. Level of Reactive Oxygen Species

The level of ROS in the MRC-5, SKOV3 and PEO1 cells after 24-h exposure to delphinidin chloride and 3-BP was assayed with 2′,7′-dichlorofluorescein diacetate (DCFDA) and dihydroethidium (DHE). Briefly, cells were seeded in a black 96-well flat clear-bottom plate at a density of 7.5 × 10^3^/well for MRC-5 fibroblasts and 1 × 10^4^ /well for cancer cells and allowed to attach at 37 °C for 24 h. After 24-h incubation, the medium was removed and replaced with a medium containing the examined substances at concentrations given previously. After incubation, the medium was removed and replaced by a 10-µM fluorescent probe (DHE or DCFDA) (100 µL/well). Stock solutions of the probes were prepared in DMSO, and working solutions were prepared in PBS. Fluorescence was measured at 490/529 nm (DCFDA) and 475/579 nm (DHE) for 2 h at 37 °C in 1-min intervals. 

### 4.7. GSH Content

The content of reduced glutathione was assayed with OPA [63]. Briefly, the cells were seeded in wells of a transparent 96-well plate at a density of 7.5 × 10^3^/well (MRC-5) or 1 × 10^4^/well (cancer cells) and allowed to attach at 37 °C for 24 h. After 24-h treatment with the tested compounds, the medium was removed, the cells were washed with PBS (150 µL per well) and then added with 60 µL/well of cold redox quenching buffer (RQB) containing 20-mM HCl, 5% TCA, 5-mM DTPA and 10-mM L-ascorbic acid. The plate was shaken for 5 minutes and centrifuged for 5 minutes at 4000 rpm. Next, the supernatant was transferred into two 96-well black plates (“+NEM” and “–NEM”; 25 µL/well). Within the “+NEM” plate, 4 µL/well of freshly prepared 7.5-mM NEM in cold RQB buffer was added. Then, 1-M phosphate buffer (pH 7.0) was added into both plates (40 µL/well), and the plates were shaken for 5 min at 700 rpm. Finally, 160 µL/well of cold 0.1-M phosphate buffer (pH 6.8) and 25 µL/well of freshly prepared 0.5% OPA in methanol were added into both plates. Both plates were incubated for 30 minutes at room temperature with constant shaking. Fluorescence was then measured at 355/430 nm. The protein contents in the cell lysates were determined according to Lowry et al. [64]. The concentration of reduced glutathione was determined by subtracting the fluorescence of the (“–NEM”) plate from the fluorescence (“+NEM”) plate and calculated with respect to the protein content.

### 4.8. Evaluation of Changes of Mitochondrial Membrane Potential (ΔΨ_m_)

Changes of mitochondrial membrane potential was evaluated using 5,5′,6,6′-tetrachloro-1,1′,3,3′-tetraethyl-imidacarbocyanine iodide, 5,5′,6,6′-tetrachloro-1,1′,3,3′-tetraethylbenzimidazolocarbocyanine iodide (JC-1) with a Mitochondrial Membrane Potential Assay Kit. In mitochondria with high ΔΨ_m_, JC-1 forms complexes with profound red fluorescence, whereas in mitochondria that exhibit low ΔΨ_m_ levels, JC-1 persists as monomers and exhibits exclusively green fluorescence.

The cells were seeded into black plates and treated as mentioned before. After 24h incubation with the studied compounds, the medium was discharged and replaced with 100x diluted JC-1 reagent in complete culture medium and incubated for 30 min in a CO_2_ incubator. Next, the plate was centrifuged at 4000 rpm for 5 min and afterwards the reagent was aspirated, and cells were washed with 150 μL/well Cell-Based Assay Buffer and centrifuged once more. After removing the supernatant, 100 μL/well of the new buffer was added. Fluorescence was measured at 540/570 nm (red fluorescence) and 485/535 nm (green fluorescence). The results were presented as a ratio of green to red fluorescence intensity ratio.

### 4.9. Mitochondrial Mass Assessment

Cells were seeded at the density of 2 × 10^5^ cells/well onto a 24-well plate and cultured as stated before. Following 24-hour exposure to the studied compounds, the cells were trypsinized, counted and transferred to separate Eppendorf tubes, and then centrifuged for 6 minutes at 3000 rpm. Subsequently the supernatant was discharged and the cells were washed with 1 mL of PBS and centrifuged again. Afterwards, 1 mL of 10 μM NAO solution in PBS was added into the samples, and incubated for 10 min in a CO_2_ incubator at 37 °C. Next, the cells were centrifuged and the pellet was washed with PBS, and resuspended in 300 μL of PBS. Each sample was transferred into a 96-well black plate (100 μL/well; 3 repetitions). Fluorescence was measured at 435/535 nm. The results were determined in relation to the cell count.

### 4.10. Cell Migration

Ovarian cancer cells and human normal fibroblasts were seeded on a 24-well plate at a density of 7 × 10^4^/well (PEO1), 6.5 × 10^4^/well (SKOV3) and 6 × 10^4^/well (MRC-5) in 700 µL of appropriate cell medium and cultured at 37 °C to reach 90–95% confluence. Then, the cells in the middle of the wells were scraped off using sterile pipette tips (100 µL), cell debris was removed by washing with medium containing 2% FBS and the tested compounds diluted with medium containing 2% FBS were added. Images of the scraped area were collected after 12 h and 24 h at 10× objective magnification using an Olympus CKX53 microscope with a U-TV0. 5XC-3 digital microscope camera. The obtained results were analyzed using ImageJ software. The percentage of scraped area closure was estimated as the ratio of area measured after 12- or 24-h incubation and the area at the beginning of the experiment. 

### 4.11. Atto-488-Phalloidin, Mitotracker and DAPI Labeling

Cells were seeded on an 8-well chamber slide (Lab-Tek™ II Chamber Slide™ System cat. no. 154534, Thermo Scientific, Waltham, MA, USA) at a density of 2.5 × 10^4^ cells/chamber and allowed to attach at 37 °C. Subsequently, PEO1 and SKOV3 cells were treated with 25-µM D, 20-µM or 40-µM 3-BP (depending on the cell line) or both substances together for 24 h. Following the treatment, the medium was removed and replaced with 200-nM solution of Mitotracker Deep Red FM (i) (cat. no. M22426, Thermo Scientific, Waltham, MA, USA) in PBS and incubated for 30 min; in a CO_2_ incubator, the cells were washed with PBS (300 μL/well) and fixed with 200-μL/well 3.7% formaldehyde for 10 min. Then, the cells were washed with PBS (200 μL/well), permeabilized with 0.1% Triton X-100 solution (200 μL/well) for 10 min and washed with PBS (700 μL/well). 

Following the treatment, the medium was removed and replaced with 150 μL/well of phalloidin working solution (ii) (prepared according to the manufacturer’s protocols) and incubated for 60 min. 

After staining i or ii, the fixed cells were washed again, 600-nM solution of DAPI in PBS was added (200 µL/well), the cells were incubated at room temperature for 60 minutes and their micrographs were taken using a Zeiss LSM 710 inverted confocal microscope (Oberkochen, Germany). The ratio of fluorescence intensity of the mitochondria and cytoskeleton (expressed as a mean gray value) was calculated using ImageJ software. The corrected total cell fluorescence (CTFT) per cell was calculated according to https://theolb.readthedocs.io/en/latest/imaging/measuring-cell-fluorescence-using-imagej.html. 

### 4.12. Apoptosis and Necrosis Assay

The levels of apoptosis and necrosis were assayed using the RealTime-Glo™ Annexin V Apoptosis and Necrosis Assay. Cells were seeded into a white 96-well plate with optical bottom, cultured and treated adequately, as stated above. After 24 h, 100 μL of the freshly prepared reagent was added to each well according to the manufacturer’s protocols. The fluorescence and luminescence were measured according to the protocols.

### 4.13. Statistical Analysis

To estimate the differences between cells treated with delphinidin chloride or 3-BP and the nontreated control, one-way ANOVA with the Least Significant Difference (LSD) post-hoc test (*n* ≥ 3 independent experiments) or Kruskal-Wallis test (*n* ≥ 6 independent experiments) was performed. In both cases, *p* ≤ 0.05 was considered statistically significant. A statistical analysis of the data was performed using the STATISTICA software package (version 13.1, StatSoft Inc., 2016, Tulsa, OK, USA). 

## 5. Conclusions

The two ovarian cell lines studied, PEO1 and SKOV3, were sensitive to 3-BP and relatively sensitive to D. Both compounds were cytotoxic to OC cells at concentrations not affecting the viability of normal fibroblasts. 3-BP and D applied together may show synergy, as exemplified in the PEO1 cells, and may potentially constitute a basis for the treatment of at least some types of OC. 

## Figures and Tables

**Table 1:** Half maximal inhibitory concentration (IC_50_) values of cell survival for the compounds studied.

Compound		IC_50_ (µM)		
	Cell Line	PEO1	SKOV3	MRC-5
3-BP		18.7	40.5	ND
Delphinidin chloride		71.5	57.5	ND
Malvidin chloride		265.1^E^	90.0	480.0 ^E^
Hispidulin		180.6	132.8 ^E^	ND
Daidzein		399.0 ^E^	132.8 ^E^	ND
Genistein		128.9 ^E^	232.1 ^E^	ND
Cyanidin chloride		106.7	ND	ND
Myricetin		196.4	ND	ND
Formononetin		ND	ND	ND
Pelargonidin		ND	ND	ND
Glycitein		ND	ND	ND
Capsaicin		ND	ND	ND

^E^ Extrapolated from data obtained in the concentration range of 1–100 µM; ND—not determined due to lack of reduction of survival in the concentration range studied. 3-BP: 3-bromopyruvic acid.

## Data Availability

Data available on request from I. Sadowska-Bartosz.
